# Scrotal wall metastasis of gastric adenocarcinoma: a rare case report and literature review

**DOI:** 10.3389/fonc.2025.1527574

**Published:** 2025-06-26

**Authors:** Yuanhao Wang, Yi Hou, Jingyu Zhang, Kunfeng Guo, Jinyu Luo, Shengnan Li, Gang Mu

**Affiliations:** Department of Urology, The Fourth People's Hospital of Shenyang, Shenyang, Liaoning, China

**Keywords:** scrotal metastasis, gastric cancer, case report, urology, cancer

## Abstract

Metastasis of gastric adenocarcinoma typically involves the liver, peritoneum, and lymph nodes, whereas scrotal wall metastasis is extremely rare, with very few cases reported in the literature. We report a case of a 60-year-old male patient who presented with right-sided scrotal swelling. The patient had previously undergone total gastrectomy and multiple chemotherapy sessions for gastric adenocarcinoma. In June 2024, the scrotal wall metastasis of gastric adenocarcinoma was confirmed. The patient underwent excision of the right scrotal lesion, spermatic cord excision, and right epididymis and testis resection. Immunohistochemical markers, such as CK20, CK7, and CDX-2, were positive, which supported the diagnosis of metastatic gastric adenocarcinoma. Follow-up three months post-surgery showed no evidence of tumor recurrence.

## 1 Case presentation

The patient, a 60-year-old male, was admitted to our hospital with a right inguinal mass that had been present for five years, and progressive right-sided scrotal swelling that had developed over the preceding two months. Since 2019, a palpable cord-like mass had gradually enlarged in the right inguinal area, but had not been given attention or treated. Beginning two months ago, the patient noticed a gradual increase in the right scrotal mass, which was non-tender, hard and swollen, and had a dragging sensation. In June 2024, the patient presented to our hospital and underwent an ultrasound-guided scrotal biopsy, which suggested a moderately differentiated adenocarcinoma with gastrointestinal origin, consistent with metastasis from gastric cancer. Immunohistochemical staining was as follows: PSA (-), Ki67 (35%), P53 (wild-type), CK20 (+), CK7 (+), TTF-1 (-), CDX-2 (+), GATA-3 (-), Vimentin (stroma +), CK19 (+), MSH2 (+), MSH6 (+), MLH1 (+), PMS2 (+), C-erbB-2 (+), CKpan (+), Villin (+),Claudin18.2 (low expression). Genetic testing results indicated the presence of KRAS p.G12V and TP53 c.993 + 2T>A mutations. Additionally, the tumor mutational burden (TMB) was 1.10 muts/Mb (classified as low level), and the microsatellite instability (MSI) status was microsatellite stable (MSS). The patient was treated in our hospital’s oncology department with a third-line regimen of albumin-bound paclitaxel plus Ramucirumab for two cycles, after which the right scrotal mass gradually reduced in size, and symptoms improved.

**Figure 1:**
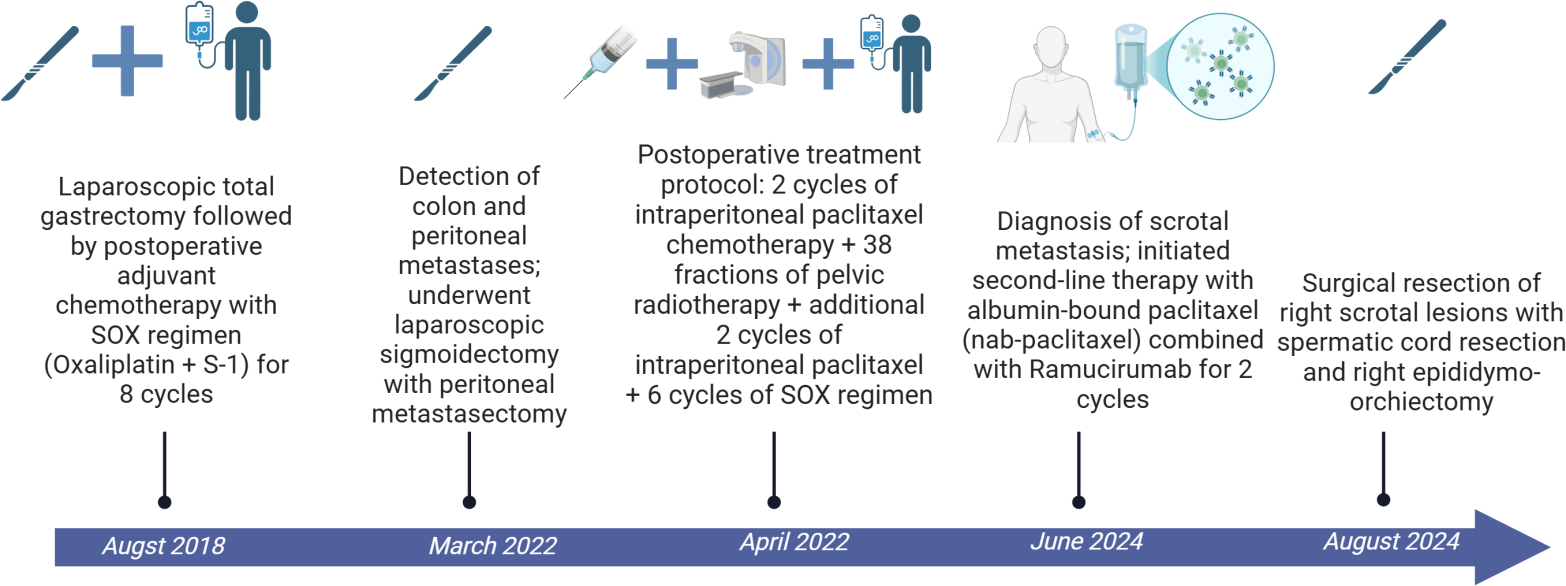
Patient treatment process.

The patient had undergone a laparoscopic total gastrectomy in August 2018, with postoperative pathology indicating a moderately-to-poorly differentiated ulcerative adenocarcinoma with nerve invasion, but no vascular invasion or lymph node metastasis (0/34 nodes). Subsequently, the patient received eight cycles of SOX (oxaliplatin plus S-1) adjuvant chemotherapy. In March 2022, follow-up revealed colonic and peritoneal metastases, and in April 2022, the patient underwent laparoscopic exploration, sigmoid colon resection, and resection of peritoneal metastases, with pathology indicating moderate differentiation of the adenocarcinoma. Postoperative treatment consisted of two cycles of intraperitoneal chemotherapy with paclitaxel, 38 sessions of pelvic radiotherapy, and another two cycles of intraperitoneal chemotherapy with paclitaxel, followed by six cycles of the SOX regimen. (**Figure 1**).

**Figure 2:**
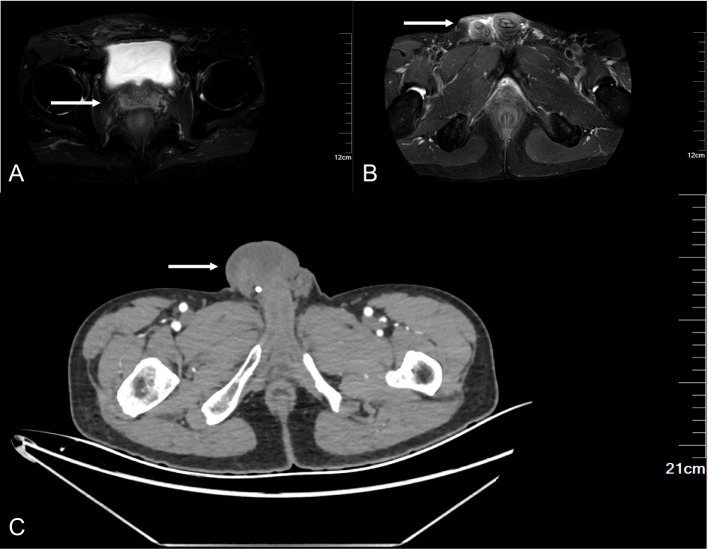
Imaging examination. **(A, B)** MRI **(C)** Enhanced CT.

After admission, the relevant examination was completed, and the tumor marker was abnormal, and the level of non-small cell lung cancer-associated antigen (CYFRA21-1) was increased by 14.38 ng/ml (normal range 0-3.3 ng/ml). Scrotal ultrasound revealed diffuse thickening (up to 1.1 cm) of the skin and subcutaneous tissue of the right scrotum, coarse echo, and increased blood vessels. Pelvic MRI revealed fluid accumulation in the right seminal vesicle and right testicular sac. Enhanced abdominal CT revealed a cord-like mass in the right groin area with bilateral hydrocele (**Figure 2**).

**Figure 3:**
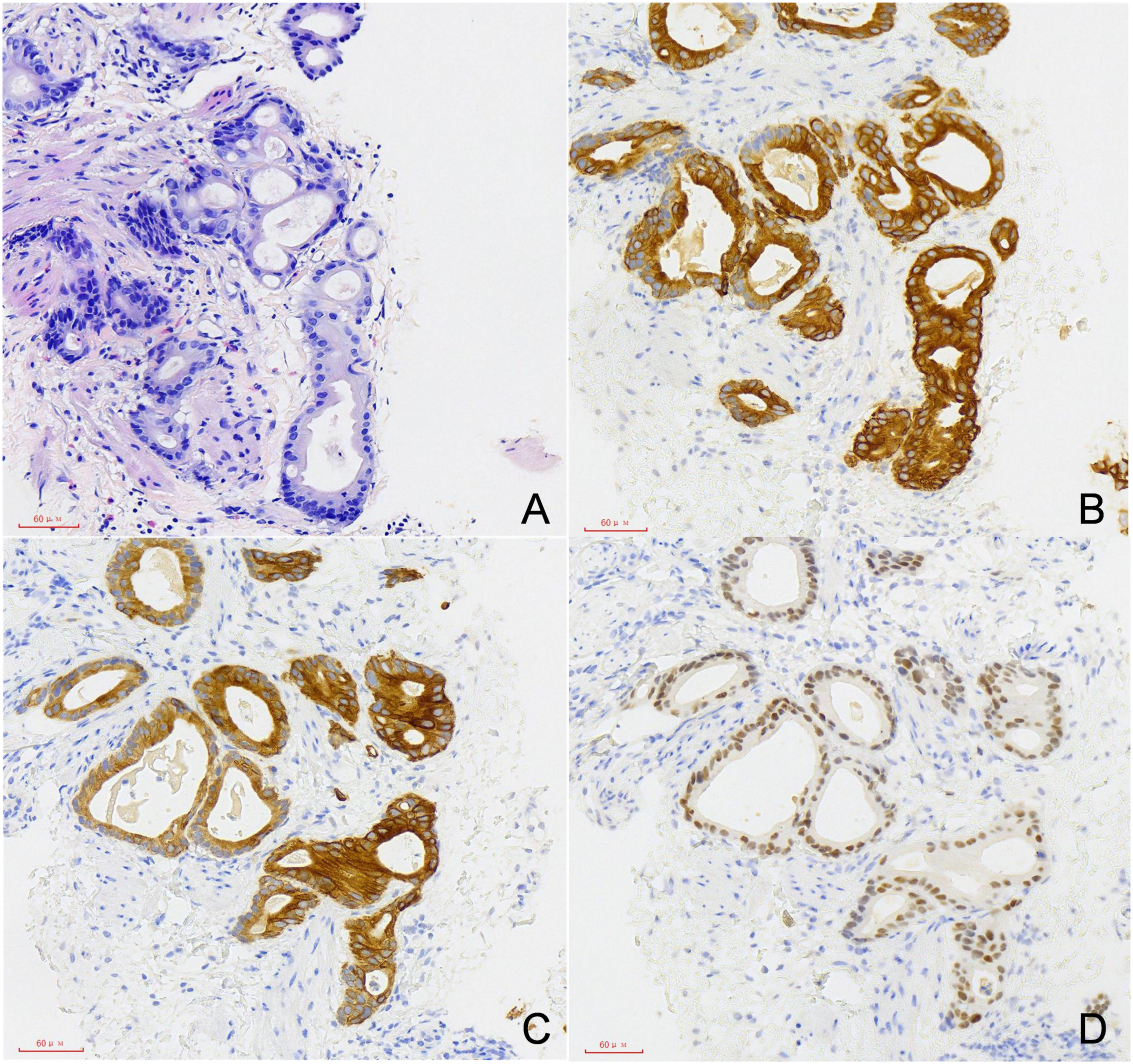
Immunohistochemical of tumor (200×). **(A)** HE **(B)** CK20 **(C)** CK7 **(D)** CDX-2.

The patient underwent right scrotal lesion resection, right spermatic cord resection, and right epididymis and testis resection in August 2024. Postoperative pathology confirmed the scrotal mass as metastatic adenocarcinoma, which had metastasized to the spermatic cord, but no testicular invasion was observed. No recurrence was observed at 3 months follow-up (**Figure 3**).

## 2 Discussion

Gastric cancer (GC) is one of the most common malignancies worldwide and the fourth leading cause of cancer mortality globally (1). Common metastatic sites for gastric cancer include the liver, peritoneum, and lymph nodes; late-stage cases may also spread to organs such as the lungs, brain, and bones, but skin metastasis is rare (2). Kim et al. (3) analyzed 14,053 cases of gastric adenocarcinoma and found that only 27 patients (0.19%) exhibited cutaneous metastases. Notably, among gastric cancer patients with cutaneous metastases, 56.9% involved the chest/abdominal walls and 54.2% affected the head/neck region, whereas scrotal involvement was exceedingly rare (4, 5). We have summarized all cases of gastric cancer with scrotal metastasis identified to date in the literature, as presented in **Table 1**. The mechanism of metastasis from stomach cancer to the spermatic cord or testis remains unclear. Several possible pathways have been proposed in the literature, such as lymphatic, vascular, hematogenous, and peritoneal routes, with the main suggested routes being the lymphatic and/or vascular pathways (18).Given the rarity of scrotal metastasis, such lesions are prone to be misdiagnosed as benign conditions, leading to delayed diagnosis. In addition to hematogenous and lymphatic routes, direct invasion along the spermatic cord structures may constitute another critical mechanism. Based on the clinical features of this case and supporting literature evidence, the potential pathways for gastric cancer metastasis to the spermatic cord can be summarized as follows:

**Table 1:** Cases of gastric cancer with scrotal metastasis.

Case	Author	Year	Age	Histology	Treatment of metastatic tumors	Prognosis
Case1 (6)	Faruk Tas	2014	69	Adenocarcinoma	Chemotherapy was combined with cisplatin, docetaxel and 5-fluorouracil.	3 months
Case2 (7)	S T Leung	2014	66	Adenocarcinoma	XELOX regimen (capecitabine combined with oxaliplatin)+FOLFOX regimen (folorin, fluorouracil combined with oxaliplatin)	Still alive at 10 months of follow-up
Case3 (8)	M Miwa	1981	21	Signet ring cell carcinoma	NA	7 months
Case4 (9)	Nobuyuki Sekita	2020	76	Adenocarcinoma	S1(Tegafur)	Still alive at 5 months of follow-up
Case5 (10)	Y Kageyama	1997	62	Adenocarcinoma	NA	NA

### 2.1 Direct invasion

Gastric cancer cells may infiltrate through the retroperitoneal space along the spermatic cord sheath or adjacent tissues. Hanash et al. (4) proposed that embryonic remnants (e.g., the processus vaginalis) could provide anatomical conduits for tumor cell descent.

### 2.2 Lymphatic metastasis

Evidence suggests overlapping lymphatic metastatic pathways between testicular and gastric malignancies. When gastric carcinoma cells obstruct the thoracic duct, the resultant retrograde lymphatic flow may facilitate tumor cell dissemination to the scrotum via the cisterna chyli and genitourinary lymphatic trunks (19).Yao et al. (5) demonstrated that approximately 30% of cutaneous metastases are associated with lymphatic dissemination, particularly when the retroperitoneal lymphatic chain is involved, allowing tumor cells to extend into the spermatic cord. Kageyama et al. (10) reported a case of gastric cancer metastasis via lymphovascular tumor thrombi within the spermatic cord, providing histopathological evidence supporting this mechanism.

### 2.3 Hematogenous metastasis

The blood supply and drainage of the spermatic cord are achieved through the coordinated function of arterial and venous systems. In the arterial system: The testicular artery, originating directly from the abdominal aorta, descends along the spermatic cord into the scrotum and is primarily responsible for oxygenated blood delivery to the testes and epididymis. The deferential artery, arising from the superior vesical artery, courses along the ductus deferens to supply surrounding tissues. The cremasteric artery, originating from the inferior epigastric artery, distributes to the cremaster muscle and external spermatic fascia, contributing to testicular positional regulation. The venous system centers around the pampiniform plexus - a plexiform network formed by a dense network of extensively anastomosing small veins that encircle the testicular artery, facilitating thermoregulation and venous return (20). Tas et al. (6) reported a case of gastric cancer with concurrent multifocal metastases to the scrotum and facial skin, providing clinical evidence supporting the plausibility of hematogenous spread.

### 2.4 Peritoneal seeding metastasis

When peritoneal metastatic lesions are located near the root of the spermatic cord, tumor cells may infiltrate the spermatic cord through local invasion. Sekita et al. (9) intraoperatively observed adhesive lesions between the peritoneum and spermatic cord, providing direct pathological evidence supporting this mechanism.

In the present case, radiographic and intraoperative findings ruled out direct invasion or peritoneal seeding. Notably, the patient had a history of peritoneal metastasis in 2022, which may have established a pre-existing lymphatic pathway for tumor dissemination. Park et al. (18) demonstrated that retroperitoneal lymphatic trunks connecting the peritoneum and spermatic cord could facilitate retrograde lymphatic spread even after peritoneal lesions regress. Residual tumor cells from prior peritoneal metastasis might have migrated along these pathways, bypassing the need for active peritoneal involvement at the time of scrotal metastasis. This hypothesis aligns with the proposed mechanism of retrograde lymphatic dissemination via the thoracic duct or genitourinary lymphatic trunks (5, 10).However, definitive evidence, such as the identification of lymphovascular tumor emboli, is required to solidify this conclusion.

This finding underscores the necessity for surgeons to maintain heightened vigilance for spermatic cord or scrotal lesions during long-term postoperative follow-up. We recommend incorporating scrotal ultrasound into routine follow-up protocols for gastric cancer patients and emphasize the importance of pathological evaluation for scrotal masses of unknown etiology. The clinical manifestation of scrotal skin metastasis is typically a painless nodule or mass without specific morphological features, making it difficult to distinguish from other benign lesions, such as sebaceous cysts, abscesses, or localized infections (11). Therefore, imaging evaluation and histopathological examination are crucial for diagnosis. Scrotal ultrasound is often the preferred imaging modality for scrotal lesions due to its high resolution and non-invasiveness, effectively differentiating abnormalities in subcutaneous tissues from testicular parenchyma. However, ultrasound has limitations in distinguishing primary malignancies from metastatic lesions, and final diagnosis relies on pathological examination and immunohistochemical markers (12). In this patient, immunohistochemical analysis showed positive expression for CK20, CK7, and CDX-2, strongly supporting the diagnosis of scrotal metastasis from gastric adenocarcinoma.

The prognosis for patients with cutaneous metastases is generally poor, especially for those with widespread visceral metastases. The median survival for gastric cancer patients with skin metastasis is typically less than three months (13, 14). However, some patients have achieved long-term survival with aggressive treatment, including surgical resection, chemotherapy, targeted therapy, and immunotherapy (15, 16). In this case, next-generation sequencing (NGS) analysis identified KRAS p.G12V and TP53 splice-site mutations, indicating sustained activation of the MAPK signaling pathway and genomic instability (21). Although low expression of Claudin18.2 restricts targeted therapy options, MEK inhibitors targeting KRAS (e.g., trametinib) and WEE1 inhibitors addressing TP53 mutations (e.g., adavosertib) may synergistically overcome drug resistance (21, 22). The MSS status and low TMB suggest avoiding immune monotherapy, while combination with anti-angiogenic agents (e.g., Ramucirumab) may enhance immune microenvironment responsiveness. Future efforts should focus on dynamic monitoring of PD-L1 expression to guide precise therapeutic interventions in immunotherapy. The recent introduction of immune checkpoint inhibitors, such as PD-1/PD-L1 inhibitors, has markedly changed the treatment landscape for advanced gastric cancer. Studies indicate that immunotherapy shows substantial efficacy in patients with high PD-L1 expression, with some cases showing long-term remission (17). Future research should focus on exploring the applications of targeted and immunotherapies for advanced gastric cancer and its rare metastatic presentations to provide more treatment options and improve prognosis.

## 3 Conclusion

In this case, the patient’s initial presentation was a painless scrotal mass, which did not receive adequate attention, illustrating a key challenge in clinical practice—the rarity of metastasis sites can lead clinicians to overlook the possibility of primary cancer, delaying diagnosis and treatment. In conclusion, scrotal metastasis from gastric adenocarcinoma is a rare and challenging clinical presentation. Raising awareness among clinicians regarding such uncommon metastatic patterns is crucial for early diagnosis and timely intervention. Despite the generally poor prognosis, prompt diagnosis and individualized, multidisciplinary treatment may provide symptom relief and improve quality of life. Future research should focus on better understanding the metastatic mechanisms of gastric cancer, particularly to unusual sites, and on developing more effective therapeutic strategies to improve overall survival in such patients.

## Data Availability

The original contributions presented in the study are included in the article/supplementary material. Further inquiries can be directed to the corresponding author.
